# The Association between *OGG1* Ser326Cys Polymorphism and Lung Cancer Susceptibility: A Meta-Analysis of 27 Studies

**DOI:** 10.1371/journal.pone.0035970

**Published:** 2012-04-23

**Authors:** Wei-Xun Duan, Rui-Xi Hua, Wei Yi, Li-Jun Shen, Zhen-Xiao Jin, Yu-Hong Zhao, Ding-Hua Yi, Wen-Sheng Chen, Shi-Qiang Yu

**Affiliations:** 1 Department of Cardiothoracic Surgery, Xijing Hospital, The Fourth Military Medical University, Xi'an, China; 2 Department of Medical Oncology, Fudan University Shanghai Cancer Center, Shanghai, China; 3 Department of Oncology, Shanghai Medical College, Fudan University, Shanghai, China; 4 Zhejiang Provincial Key Laboratory of Medical Genetics, Wenzhou Medical College, Wenzhou, China; University of Navarra, Spain

## Abstract

**Background:**

Numerous studies have investigated association of *OGG1* Ser326Cys polymorphism with lung cancer susceptibility; however, the findings are inconsistent. Therefore, we performed a meta-analysis based on 27 publications encompass 9663 cases and 11348 controls to comprehensively evaluate such associations.

**Methods:**

We searched publications from MEDLINE and EMBASE which were assessing the associations between *OGG1* Ser326Cys polymorphism and lung cancer risk. We calculated pooled odds ratio (OR) and 95% confidence interval (CI) by using either fixed-effects or random-effects model. We used genotype based mRNA expression data from HapMap for SNP rs1052133 in normal cell lines among 270 subjects with four different ethnicities.

**Results:**

The results showed that individuals carrying the Cys/Cys genotype did not have significantly increased risk for lung cancer (OR = 1.15, 95% CI = 0.98–1.36) when compared with the Ser/Ser genotype; similarly, no significant association was found in recessive, dominant or heterozygous co-dominant model (Ser/Cys vs. Cys/Cys). However, markedly increased risks were found in relatively large sample size (Ser/Ser vs. Cys/Cys: OR = 1.29, 95% CI = 1.13–1.48, and recessive model: OR = 1.19, 95% CI = 1.07–1.32). As to histological types, we found the Cys/Cys was associated with adenocarcinoma risk (Ser/Ser vs. Cys/Cys: OR = 1.32, 95% CI = 1.12–1.56; Ser/Cys vs. Cys/Cys: OR = 1.19, 95% CI = 1.04–1.37, and recessive model OR = 1.23, 95% CI = 1.08–1.40). No significant difference of *OGG1* mRNA expression was found among genotypes between different ethnicities.

**Conclusions:**

Despite some limitations, this meta-analysis established solid statistical evidence for an association between the *OGG1* Cys/Cys genotype and lung cancer risk, particularly for studies with large sample size and adenocarcinoma, but this association warrants additional validation in larger and well designed studies.

## Introduction

Cancer is recognized as the leading cause of death in economically developed countries and the second leading cause of death in developing countries. It has been estimated that approximately 12.7 million cancer cases and 7.6 million cancer deaths have been occurred in 2008. Lung cancer was the most commonly diagnosed type of cancer as well as the leading cause of cancer death in males in 2008. Globally, lung cancer accounts for 13% (1.6 million) of the total cases and 18% (1.4 million) of the deaths [1]. Cigarette smoking is the well known risk factor for lung cancer, which accounts for 80% of the worldwide lung cancer burden in males and at least 50% of the burden in females [2]. Tobacco smoke contains multiple carcinogens that are known to chemically modify of genomic DNA [3] and further lead to genetic mutations [4].

DNA repair genes play a crucial role in maintaining the stability and integrity of genomic DNA. In humans, more than 130 genes are involved in the five major DNA repair pathways, one of which is base excision repair (BER) pathway [5]. The BER pathway repairs lesions involving modifications to the DNA bases, including lesions generated by reactive oxygen species. The specificity of BER is supported by DNA glycosylases, which have precise substrate specificities. In mammalian cells there are four major DNA glycosylases including oxoguanine DNA glycosylase (OGG1), which primarily recognizes 8-oxodG but also active on other oxidized purines [6].

The 8-oxoguanine DNA glycosylase (*OGG1*) gene, located at chromosome 3p26.2, encodes the enzyme responsible for the excision of 8-oxoguanine, a mutagenic base byproduct which occurs as a result of exposure to reactive oxygen species. It catalyzes the cleavage of the glycosylic bond between the modified base and the sugar moiety, leaving an abasic apurinic/apyrimidinic site in DNA; the resulting site is then incised, followed by completing of repair with successive actions of a phosphodiesterase, a DNA polymerase and a DNA ligase [7], [8]. The *OGG1* is highly polymorphic, and a number of single nucleotide polymorphisms (SNPs) have been identified [9]–[12], with at least 231 reported SNPs in the gene region (http://www.ncbi.nlm.nih.gov/projects/SNP). However, only few of these reported SNPs are potentially functional and been studied for their associations with cancer susceptibility. For *OGG1*, there are twenty five SNPs that reportedly change amino acid of the protein but only Ser326Cys (rs1052133) was extensively investigated for its association with cancer risk, in particular for lung cancer. Because the results from these studies were inconsistent [9], [13]–[25], we performed a meta-analysis of the published reports to further evaluate the association of *OGG1* Ser326Cys SNPs with the risk of lung cancer.

## Materials and Methods

### Identification and eligibility of relevant studies

Studies included in this meta-analysis were to meet the following criteria: (a) evaluating the association between *OGG1* Ser326Cys and cancer risk, (b) using a case-control design, (c) providing sufficient information to estimate odds ratios (ORs) and their 95% confidence intervals (CIs).

We searched the electronic literature MEDLINE and EMBASE databases for all relevant articles using the search terms: “*OGG1*, *HMMH*, *MUTM*, *OGH1* or *hOGG1*”, “variant or variation or polymorphism” and “lung cancer” (last search was updated on Nov 30, 2011). All eligible studies were retrieved, and their bibliographies were manually checked for other relevant publications. Review articles and bibliographies of other relevant studies identified were hand-searched as well to search for additional eligible studies. Only published studies with full-text articles in English were included. If more than one article was published using the same patient population, only the latest or the largest study would be used in this meta-analysis. Two authors (Wei-Xun Duan and Rui-Xi Hua) independently assessed the articles for compliance with the inclusion criteria, and any disagreement was resolved by discussions till consensus was reached. In addition, investigations departure from Hardy-Weinberg equilibrium (HWE) was excluded from the final analysis.

### Data extraction

The following information was collected from each study: first author's surname, year of publication, ethnicity of the study population, cancer types, histological types, source used for controls, total number of cases and controls, genotype methods and numbers of cases and controls with the Ser/Ser, Ser/Cys, and Cys/Cys genotypes for *OGG1*. For those studies that included subjects of different ethnic groups, genotypes data were extracted separately for each of ethnic groups, categorized as Caucasians, Asians, Africans or Mixed which contained more than one ethnic group.

### Genotype and gene expression correlation analysis

The data on *OGG1* Ser326Cys (rs1052133C>G) genotype and transcript (mRNA) expression levels were available by SNPexp online tool (http://app3.titan.uio.no/biotools/help.php?app=snpexp) [26]. The genotyping data for *OGG1* were derived from the HapMap phase II release 23 data set consisting of 3.96 million SNP genotypes from 270 individuals from four populations (CEU: 90 Utah residents with ancestry from northern and western Europe; CHB: 45 unrelated Han Chinese in Beijing; JPT: 45 unrelated Japanese in Tokyo; YRI: 90 Yoruba in Ibadan, Nigeria) [27]. The transcript (mRNA) expression data were detected by using genome-wide expression arrays (47294 transcripts) from EBV-transformed lymphoblastoid cell lines from the same 270 individuals [28].

### Statistical methods

The strength of association between *OGG1* Ser326Cys and lung cancer risk was assessed by calculating ORs with the corresponding 95% CIs. For *OGG1* Ser326Cys, the pooled ORs were also performed for additive (Ser/Ser vs. Cys/Cys and Ser/Cys vs. Cys/Cys), recessive model (Ser/Ser+Ser/Cys vs. Cys/Cys), and dominant model (Ser/Ser vs. Ser/Cys+Cys/Cys). The homogeneity assumption was verified by Chi square-based Q-test. If the studies were found to be homogeneous (with *P*>0.10 for the Q test), the pooled OR estimate of all studies would be calculated by the fixed-effects model (the Mantel–Haenszel method) [29]. If homogeneity could not be assumed, a random-effects model (the DerSimonian and Laird method) would be used [30]. Subgroup analyses were performed by cancer type, ethnicity, study design and sample size. To verify the potential publication bias, a standard error of log (OR) for each study was plotted against its log (OR). Funnel plot asymmetry was assessed by Egger's linear regression test [31]. To assess the effect of individual studies on the overall risk of cancers, sensitivity analyses were performed by excluding each study individually and recalculating the ORs and the 95% CIs. The mRNA expression levels between two strata were assessed by using Student's t test. The transcript expression level trends by genotypes were evaluated by using General linear model. This meta-analysis was performed by using the software STATA version 11.0 (Stata Corporation, College Station, TX) and SAS software (version 9.1; SAS Institute, Cary, NC). All the *P* values were two-sided, and a *P*<0.05 was considered statistically significant.

## Results

### Study characteristics

**Figure 1:**
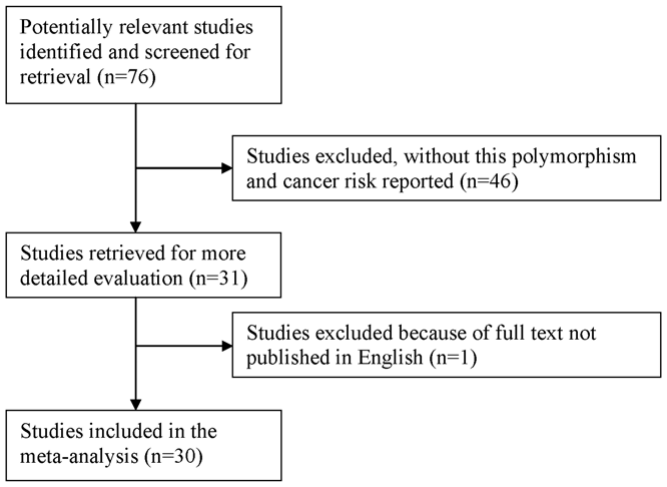
Flow chart of Included Studies.

As shown in Figure 1, a total of 76 published records were retrieved, of which 45 were excluded after the abstract was found to be irrelevant, and one paper was excluded since they published in Korean [32]. A total of 30 case-control studies met the inclusion criteria [9], [13]–[25], [33] and were included in the meta-analysis (Table 1). The study by Bonner et al. [33] was excluded in the final analysis because they used the same samples as a previous article [34]. The distribution of genotypes for the *OGG1* polymorphism in the controls of all studies was consistent with that expected from the HWE, except for three studies [23], [35], [36]. Chang et al. [19] evaluate the differences in genetic contribution to lung cancer risk in Latinos and African-American ethnic groups, so this study was separated in two. Overall, 27 studies with 9663 cases and 11348 controls investigating the *OGG1* Ser326Cys SNP were included in this meta-analysis. The study of Klinchid et al. [20] was included only in the calculation of the dominant model, because the genotype distribution was not presented in sufficient detail. Of the 27 studies, sample sizes ranged from 45 to 2155, in which eight studies focused on non-small cell lung cancer (NSCLC) and nineteen on mixed lung cancers. There were eleven studies on Caucasians, twelve studies on Asians, two studies on Africans and two on mixed ethnicity. Almost all of the cases were histologically confirmed. Controls were mainly matched for sex and age. Of all the studies, twelve were population-based, fifteen were hospital-based; three studies with sample size less than 100, nineteen studies with sample size between 100 to 500, and five studies with sample size more than 500. For available histological types, six studies with small cell lung cancer (SCLC), seven with squamous cell carcinoma (SCC), ten with adenocarcinoma (ADC), one with large cell carcinoma (LCC), six with NSCLC thus histological type details not available, and twelve with lung cancer but details not available that were considered as mixed.

**Table 1:** Characteristics of studies included in the meta-analysis.

Surname	Year	Country	Ethnicity	Cancer type	Cases/controls	Control source	Genotype method	MAF	H-W
Khono	1998	Japan	Asian	Mixed	45/42	PB	PCR-SSCP	0.40	0.939
Sugimura	1999	Japan	Mixed	Mixed	241/197	HB	PCR-SSCP	0.41	0.082
Wikman	2000	Germany	Caucasian	NSCLC	105/105	HB	PCR-RFLP	0.22	0.067
Ito	2002	Japan	Asian	ADC	138/240	HB	PCR-CTPP	0.47	0.837
Le	2002	USA	Mixed	Mixed	298/405	PB	PCR-RFLP	0.35	0.350
Sunaga	2002	Japan	Asian	ADC	198/152	HB	PCR-RFLP	0.45	0.126
Lan	2004	China	Asian	Mixed	118/109	PB	SNP500	0.33	0.232
Park	2004	USA	Caucasian	Mixed	179/350	HB	PCR-RFLP	0.15	0.857
Vogel	2004	Denmark	Caucasian	Mixed	256/269	PB	real-time PCR	0.24	0.237
Hung	2005	European	Caucasian	SCC, ADC	2155/2163	HB	TaqMan	0.20	0.215
Khono	2006	Japan	Asian	ADC	1097/394	HB	pyrosequencing	0.45	0.627
Loft	2006	Denmark	Caucasian	Mixed	251/261	PB	real-time PCR	0.24	0.200
Sorensen	2006	Denmark	Caucasian	Mixed	431/796	PB	real-time PCR	0.22	0.258
Matullo	2006	European	Caucasian	Mixed	116/1094	PB	DHPLC	0.22	0.901
De Ruyck	2007	Belgium	Caucasian	Mixed	110/110	HB	PCR-RFLP	0.25	0.176
Hatt	2008	Denmark	Caucasian	Mixed	158/164	PB	TaqMan	0.25	0.536
Karahalil	2008	Turkey	Caucasian	Mixed	165/250	PB	PCR-RFLP	0.33	0.546
Chang	2009	Taiwan	Asian	Mixed	1096/997	HB	MassARRAY	0.60	0.741
Miyaishi	2009	Japan	Asian	Mixed	108/121	HB	PCR-RFLP	0.45	0.271
Okasaka	2009	Japan	Asian	Mixed	515/1030	PB	TaqMan	0.49	0.070
Chang	2009	USA	African	Mixed	112/296	PB	Illumina	0.32	0.691
Chang	2009	USA	African	Mixed	254/280	PB	Illumina	0.15	0.521
Klinchid	2009	Thailand	Asian	NSCLC	76/75	HB	diASA-AMP		
Janik	2011	Poland	Caucasian	NSCLC	88/79	HB	PCR-MSSCP	0.15	0.542
Kohno	2011	Japan	Asian	SCC	377/325	HB	pyrosequencing	0.45	0.704
Li	2011	China	Asian	Mixed	395/443	HB	PCR-CTPP	0.62	0.329
Qian	2011	China	Asian	NSCLC	581/601	HB	TaqMan	0.55	0.592
Liang	2005	China	Asian	SCC, ADC	227/227	HB	diASA-AMP	0.61	**0.043**
Zienolddiny	2006	Norway	Caucasian	NSCLC	326/386	PB	APEX	0.35	**0.000**
Liu	2010	Taiwan	Asian	Mixed	358/716	HB	PCR-RFLP	0.64	**0.004**

HB, Hospital based; PB, Population based; SCC, Squamous Cell Carcinoma; NSCLC, non-small cell lung cancer; ADC, Adenocarcinoma; PCR-SSCP, PCR-single strand conformation polymorphism; PCR-RFLP, PCR-restriction fragment length polymorphisms; PCR-CTPP, polymerase chain reaction with confronting two-pair primers; diASA-AMP, di-allele-specific amplification with artificially modified primers; DHPLC, denaturing high performance liquid chromatography.

### Meta-analysis results

**Figure 2:**
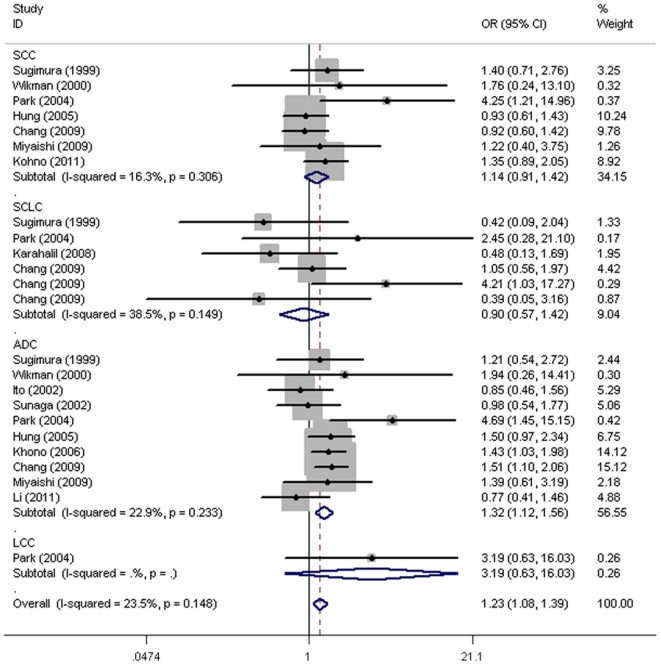
ORs of lung cancer with definite histological types associated with the *OGG1* Ser/Ser genotype compared with the Cys/Cys genotype. For each study, the estimates of OR and its 95% CI are plotted with a box and a horizontal line. ⋄, pooled ORs and its 95% CIs.

The overall results suggested there was no significant association between *OGG1* Ser326Cys and risk of lung cancer (Ser/Ser vs. Cys/Cys: OR = 1.15, 95% CI = 0.98–1.36; Ser/Cys vs. Cys/Cys: OR = 1.09, 95% CI = 0.95–1.25), recessive (OR = 1.11, 95% CI = 0.97–1.28) or dominant model (OR = 1.09, 95% CI = 0.98–1.21) (Table 2). In the subgroup analysis by sample size, a statistically significant association was found for studies with large sample size (Ser/Ser vs. Cys/Cys: OR = 1.29, 95% CI = 1.13–1.48; Ser/Ser+Ser/Cys vs. Cys/Cys: OR = 1.19, 95% CI = 1.07–1.32), and a relative small sample size (<100) for dominant model (OR = 1.79, 95% CI = 1.08–2.98) which just included three studies. Further stratification by cancer type, ethnicity, and source of controls all yielded no statistically significant estimates. In the histological type subgroup analysis with more details, the *OGG1* Ser326Cys allele was significantly associated with risk of ADC (Ser/Ser vs. Cys/Cys: OR = 1.32, 95% CI = 1.12–1.56; Ser/Cys vs. Cys/Cys: OR = 1.19, 95% CI = 1.04–1.37) and recessive model (OR = 1.23, 95% CI = 1.08–1.40), but not with cancers of the SCC, SCLC and LCC (Table 3, Figure 2).

**Table 2:** Meta-analysis of the association between *OGG1* Ser326Cys polymorphism and lung cancer risk.

Variables	No. of studies	Homozygous	*P*_het_ a	Heterozygous	*P*_het_ a	Recessive	*P*_het_ a	Dominant	*P*_het_ a
		Ser/Ser vs. Cys/Cys		Ser/Cys vs. Cys/Cys		(Ser/Ser+Ser/Cys) vs. Cys/Cys		Ser/Ser vs. (Ser/Cys+Cys/Cys)	
All	27	1.15 (0.98–1.36)	0.001	1.09 (0.95–1.25)	0.010	1.11 (0.97–1.28)	0.002	1.09 (0.98–1.21)	<0.001
Cancer type									
NSCLC	8	1.29 (0.99–1.69)	0.118	1.14 (0.85–1.55)	0.054	1.18 (0.90–1.54)	0.029	1.20 (0.99–1.45)	0.125
Mixed	19	1.09 (0.89–1.34)	0.002	1.08 (0.92–1.26)	0.016	1.09 (0.92–1.29)	0.006	1.05 (0.93–1.19)	0.001
Ethnicity									
Caucasian	11	1.12 (0.78–1.62)	0.014	1.08 (0.79–1.47)	0.107	1.11 (0.79–1.55)	0.028	1.05 (0.88–1.25)	0.001
Asian	12	1.15 (0.92–1.43)	0.004	1.04 (0.89–1.22)	0.030	1.07 (0.91–1.27)	0.007	1.15 (0.98–1.35)	0.018
African	2	1.00 (0.54–1.84)	0.802	0.98 (0.53–1.82)	0.423	1.01 (0.56–1.83)	0.653	1.12 (0.82–1.52)	0.279
Mixed	2	1.48 (0.96–2.27)	0.224	1.73 (1.23–2.43)	0.368	**1.61 (1.14–2.28)**	0.284	1.01 (0.79–1.29)	0.334
Source of controls									
Population	12	1.15 (0.94–1.40)	0.335	1.09 (0.88–1.36)	0.221	1.14 (0.94–1.38)	0.273	1.08 (0.97–1.19)	0.617
Hospital	15	1.20 (0.94–1.54)	<0.001	1.11 (0.92–1.33)	0.005	1.13 (0.93–1.38)	<0.001	1.10 (0.93–1.31)	<0.001
Sample size									
<100	3	4.46 (0.29–68.84)	0.021	3.88 (0.41–36.47)	0.059	4.33 (0.33–57.22)	0.024	**1.79 (1.08–2.98)**	0.302
100–500	19	1.05 (0.83–1.34)	0.002	1.01 (0.82–1.25)	0.010	1.04 (0.84–1.29)	0.002	1.04 (0.91–1.19)	0.002
>500	5	**1.29 (1.13–1.48)**	0.920	1.05 (0.81–1.37)	0.842	**1.19 (1.07–1.32)**	0.953	1.12 (0.96–1.31)	0.046

a *P* value of the Q-test for heterogeneity test.

**Table 3:** Meta-analysis of the association between *OGG1* Ser326Cys polymorphism and lung cancer with definite histological types.

Variables	No. of studies	Homozygous	*P*_het_ a	Heterozygous	*P*_het_ a	Recessive	*P*_het_ a	Dominant	*P*_het_ a
		Ser/Ser vs. Cys/Cys		Ser/Cys vs. Cys/Cys		(Ser/Ser+Ser/Cys) vs. Cys/Cys		Ser/Ser vs. (Ser/Cys+Cys/Cys)	
Histological types									
SCLC	6	0.90 (0.57–1.42)	0.149	0.98 (0.67–1.44)	0.696	0.97 (0.68–1.39)	0.357	1.07 (0.67–1.72)	0.014
SCC	7	1.14 (0.91–1.42)	0.306	1.16 (0.96–1.41)	0.163	1.14 (0.95–1.38)	0.143	1.04 (0.84–1.28)	0.101
ADC	10	**1.32 (1.12–1.56)**	0.233	**1.19 (1.04–1.37)**	0.186	**1.23 (1.08–1.40)**	0.248	1.13 (0.93–1.38)	0.014
LCC	1	3.19 (0.63–16.03)	-	2.18 (0.41–11.69)	-	2.85 (0.58–14.03)	-	1.61 (0.76–3.42)	-
NSCLC	6	**1.34 (1.03–1.75)**	0.065	1.11 (0.89–1.39)	0.155	1.18 (0.95–1.46)	0.086	1.25 (0.97–1.61)	0.147
Mixed	12	**1.24 (1.02–1.49)**	0.364	1.20 (1.00–1.43)	0.320	**1.24 (1.05–1.46)**	0.307	1.08 (0.96–1.22)	0.371

SCLC, Small cell lung cancer; SCC, Squamous Cell Carcinoma; ADC, Adenocarcinoma; LCC, Large cell carcinoma; NSCLC, non-small cell lung cancer.

a *P* value of the Q-test for heterogeneity test.

### The mRNA expression by genotypes

The mRNA expression level of *OGG1* by the genotypes of four ethnicities is shown in Table 4. We did not find any mRNA expression difference between different genotypes among the four different ethnicities. Thus, mRNA expression level was a little higher though no significant difference for rs1052133G variant allele was found in Asian populations. No trend of transcript expression levels by genotypes was found for *OGG1*.

**Table 4:** *OGG1* mRNA expression by the genotypes of SNPs, using data from the HapMap^a^.

Population	Genotypes	No.	Mean ± SD	*P* b	*P*_trend_ c
**CHB**	CC	11	7.84±0.20		0.267
	CG	23	7.94±0.26	0.280	
	GG	11	7.96±0.29	0.264	
	CG/GG	34	7.95±0.26	0.234	
**JPT** d	CC	8	7.67±0.18		0.125
	CG	26	7.80±0.18	0.084	
	GG	10	7.81±0.16	0.102	
	CG/GG	36	7.80±0.17	0.057	
**CEU** d	CC	55	7.87±0.31		0.283
	CG	26	7.89±0.28	0.781	
	GG	6	7.63±0.18	0.082	
	CG/GG	32	7.84±0.28	0.689	
**YRI** d	CC	65	7.80±0.23		0.753
	CG	20	7.77±0.20	0.568	
	GG	3	7.83±0.20	0.869	
	CG/GG	23	7.78±0.19	0.637	
**Asian** d	CC	19	7.77±0.21		0.106
	CG	49	7.86±0.23	0.117	
	GG	21	7.89±0.24	0.102	
	CG/GG	70	7.87±0.23	0.083	
**All** d	CC	139	7.82±0.26		0.620
	CG	95	7.85±0.24	0.420	
	GG	30	7.83±0.24	0.883	
	CG/GG	125	7.85±0.24	0.468	

a Genotyping data and mRNA expression levels for *OGG1* Ser326Cys by genotypes obtained from the HapMap phase II release 23 data from EBV-transformed lymphoblastoid cell lines from 270 individuals. CHB: 45 unrelated Han Chinese in Beijing; JPT: 45 unrelated Japanese in Tokyo; CEU: 90 Utah residents with ancestry from northern and western Europe; YRI: 90 Yoruba in Ibadan, Nigeria.

b Two-side Student's *t* test within the stratum.

c *P* values for the trend test of *OGG1* mRNA expression among 3 genotypes for each SNP from a general linear model.

d There were missing data because genotyping data were not available.

### Publication bias

No publication bias was detected for *OGG1* Ser326Cys (the Egger's test, Ser/Ser vs. Cys/Cys: *P* = 0.944, Ser/Cys vs. Cys/Cys: *P* = 0.987, recessive model: *P* = 0.892, dominant model: *P* = 0.217).

## Discussion

It is well recognized that individual susceptibility to cancer varies, even after exposure to the same environment. Therefore, it has been suggested that genetic variation, such as SNPs of genes is involved in carcinogenesis. We conducted a meta-analysis of published studies to evaluate the association between the *OGG1* Ser326Cys polymorphism and lung cancer risk because no such up-to-date meta-analysis including histological types has been published to date. We found no statistical evidence of an overall effect of the Ser326Cys polymorphism on lung cancer risk in either recessive or dominant effect models. Compared with the Ser/Ser genotype, the variant Cys/Cys genotype was not significantly associated with overall lung cancer risk in all subjects from 27 eligible studies included in the analysis. In a previous meta-analysis with 17 studies consist of 6375 cases and 6406 controls, significantly increased risks were found among Asian subjects in a dominant model, and lung cancer risk associated with the *OGG1* Cys/Cys genotype was significantly increased in population-based studies [9]. However, these were not found in ours, may be attributed to a larger sample size.

Previous studies demonstrated that genetic variation in *OGG1* affects cancer susceptibility; the frequency of the *OGG1* 326Cys allele was found to be significantly higher in patients when compared with controls [22], [24], [25], [37], [38]; however, this association was not replicated by other studies [9], [13]–[17]. Overall, we did not found that individuals carrying the Cys/Cys genotype had significantly increased risk of lung cancer when compared with the Ser/Ser genotype, no significant association with lung cancer risk was found in dominant model, recessive model and heterozygous co-dominant model (Ser/Cys vs. Cys/Cys). However, markedly increased risks were found in relatively large sample size, and this hinted us that in the future studies, only the studies with large sample size would be reliable. In the histological type subgroup analysis, the *OGG1* Ser326Cys allele was significantly associated with risk of ADC, but not with cancers of the SCC, SCLC and LCC. These may be attributed to the tumor specificity.

Mambo et al. [39] analyzed the expression of *hOGG1* mRNA in 18 lung cancer and three normal cell lines and found *hOGG1* was over expressed in most cell lines, 2/18 (11.1%) showed a lower *hOGG1* mRNA and protein expression (∼80% decrease) relative to normal cell lines, indicating 8-Hydroxyguanine repair defects in certain lung cancers. When we compared the mRNA expression levels of *OGG1* Ser326Cys by the genotypes of four different ethnicities, no difference or trend was found. Lung cancer is known to be a complex and multifactorial disease, gene-gene and gene-environment interactions both contribute greatly to the occurrence of this disease, a single nucleotide variation may be insufficient to alter the *OGG1* mRNA expression especially the ones in the coding regions which just lead to amino acid alteration.

Though we included the latest data, there are several limitations in this meta-analysis must also be considered. First, lack of the original data of lung cancer histological types limited our further evaluation of histological types and genotypes interactions. Second, lack of the original data limited our further evaluation of potential gene-gene and gene-environment interactions. Third, lack of information on disease status, genotypes, and well-documented smoking status may also influence the results. Fourth, most of the studies except for five [17], [21], [22], [40], [41] had a relatively small sample sizes (<500 cases and controls). Finally, the studies included in the analysis have used more than ten different genotyping methods that had different quality control issues.

In conclusion, this meta-analysis found that the *OGG1* 326Cys/Cys genotype was not associated with significantly increased risk of lung cancer. However, given the relatively limited lung cancer histological types and sample size, Cys/Cys was associated with adenocarcinoma risk. However, further studies are warranted to validate the association between the *OGG1* Ser326Cys polymorphism and lung cancer risk with larger sample size and more detailed histological types.
